# A new species of *Amara* (Coleoptera, Carabidae, Zabrini) from Sichuan Province, China, with additional records for other *Amara* species from the region

**DOI:** 10.3897/zookeys.254.4223

**Published:** 2012-12-21

**Authors:** Fritz Hieke, David H. Kavanaugh, Hongbin Liang

**Affiliations:** 1 Museum für Naturkunde der Humboldt Universität zur Berlin, Institut für Systematische Zoologie, Invalidenstrasse 43, D-10115 Berlin, Germany; 2 Department of Entomology, California Academy of Sciences, San Francisco, California 94118, U.S.A.; 3 Key Laboratory of Zoological Systematics and Evolution, Institute of Zoology, Chinese Academy of Sciences, Beijing 100101, China

**Keywords:** Coleoptera, Carabidae, Zabrini, *Amara*, new species, China, Sichuan, Yunnan

## Abstract

A new species, *Amara (Bradytulus) shalulishanica* Hieke & Kavanaugh, **sp. n.** (type locality: Haizishan Yakou, 29.47366°N, 100.21921°E, 4623 m, Shalulishan, Zhuosang Township, Litang County, Sichuan Province, China) is described and diagnosed. Additional records are provided for 16 other *Amara* species, each of which represents one of five different geographical distribution types, which are discussed.

## Introduction

Over the ten-year period from 1998 to 2007, two of us (DHK and HBL) had the opportunity to collect extensively in western Yunnan Province, China, as part of a multidisciplinary, international project to inventory the biodiversity of the Gaoligongshan (Gaoligong Mountains). This project also provided the opportunity for us to collect in a few other areas while traveling between Beijing and the study area itself, including most notably one fast traverse in 2007 across several of the mountain ranges that represent the dissected southern edge of the Tibetan Plateau in western Sichuan and northeastern Yunnan Provinces. In total, more than 2,000 specimens representing species of the carabid beetle genus *Amara* Bonelli were collected in the course of this work. Specimens collected in the Gaoligonshan region will form the basis of a separate treatment of the Zabrini of that region (in preparation), which will include a key to the species in that fauna and details of geographical and habitat distributions for each of the included species.

In this contribution, we report on *Amara* species records for specimens collected outside of the Gaoligong region, including those representing one species new to science. That new species is described and a discussion of diagnostic features facilitating discrimination of its adults from those of similar species is provided. One of us (FH) is currently engaged in a comprehenive re-evaluation of subgeneric limits and relationships among *Amara* species, so we refrain from providing a key to subgenera of *Amara* or to species likely related to our new species pending results of that analysis.

## Materials and methods

This contribution is based on the study of 279 *Amara* specimens, mainly from Sichuan and Yunnan Provinces. All of these specimens are deposited in the collections of CAS, IZCAS, or ZMHB.

Abbreviations for collections cited in this study follow Evenhuis (2012) as far as possible and include:

**BMNH** British Museum (Natural History), London, United Kingdom

**CAS** California Academy of Sciences, San Francisco, U.S.A.

**CBAL** Collection of A. Baliani, in MCSNG

**CCHA** Collection of M. de Chaudoir, in MNHN

**CFAC** Collection of S. Facchini, Torino, Italy

**CHEI** Collection of W. Heinz, Schwanfeld, Germany

**CJED** Collection of A. Jedlička, in NMPC

**CMEY** Collection of P. Meyer, Darmstadt, Germany

**CSCI** Collection of R. Sciaky, Milan, Italy

**CWRA** Collection of D. Wrase, Berlin, Germany

**DEI** Deutsches Entomologisches Institut, Eberswalde, Germany

**FRSDD** Forest Research Institute, Dehra Dun, India

**IZCAS** National Zoological Museum of China, Institute of Zoology, Beijing, China

**MCSNG** Museo Civico di Storia Naturale, Genoa, Italy

**MGFT** Museum G. Frey, Tutzing, in NHMB

**MNHN** Muséum National d’Histoire Naturelle, Paris, France

**NHMB** Naturhistorisches Museum, Basel, Switzerland

**NMPC** National Museum (Natural History), Prague, Czech Republic

**RMNH** Rijksmuseum van Natuurlijke Historie, Leiden, Netherlands

**SNF** Natur-Museum und Forschungs-Institut Senckenberg, Frankfurt am Main, Germany

**ZIN** Zoological Institute Academy of Sciences, St. Petersburg, Russia

**ZMHB** Museum für Naturkunde an der Humboldt-Universität, Berlin, Germany

**ZSM** Zoologische Sammlung des Bayerischen Staates, Munich, Germany

The only measurement recorded is that of body length, taken as a single measure along the midline from the anterior margin of the labrum to the apex of the longer elytron. Information presented on the overall geographical distributions of species is based on the Catalogue of Palaearctic species (Hieke 2003a) and on data about these species gleaned from specimens in the collection at ZMHB.

## New species

### Amara (Bradytulus) shalulishanica

Hieke & Kavanaugh sp. n.

urn:lsid:zoobank.org:act:DEF0D9F5-F136-4561-A0AC-ADE1ABBA9EB7

http://species-id.net/wiki/Amara_shalulishanica

Figs 1 2

#### Type material.

Holotype, a male, in IZCAS, labeled “CHINA, Sichuan, Litang County, Zhuosang Township, Shalulishan, Haizishan Yakou, 29.47366°N, 100.21921°E”/ “4623 m, 16 September 2007, Stop # 2007-041, D. H. Kavanaugh & H. B. Liang”/ “Holotype *Amara shalulishanica* Hieke and Kavanaugh” [red label]. Paratypes: Total 4 specimens, 1 male and 1 female in CAS, 1 female in IZCAS and 1 male in ZMHB, all with same label data as holotype, except third label reading “Paratype *Amara shalulishanica* Hieke and Kavanaugh” [yellow label]. Type locality: China, Sichuan Province, Litang County, Zhuosang Township, Shalulishan, Haizishan Yakou [29.47366°N, 100.21921°E].

#### Diagnosis.

Adults of *Amara shalulishanica* sp. n. have all the features of other members of subgenus *Bradytulus* Tschitschérine (1894), namely: pronotum with greatest width at or slightly anterior to mid-length; prosternum of male without a punctate fovea at middle; prosternal intercoxal process unmargined and apically asetose; mesofemora bisetose ventrally; mesotibiae of the male with a distinct subapical tooth (seen also in *Curtonotus* males) on medial margin and a brush-like patch of setae ventrally in apical one-fourth. The absence of an apical hook from the right paramere of male genitalia is shared with males of most other *Bradytulus* species.

No other species of subgenus *Bradytulus* is known from the Shalulishan (Shaluli Mountains) of Sichuan Province, China. Most species of this subgenus live in the Himalaya Mountains and/or Xizang Province (Tibet) (Hieke 2003b). *Amara thibetana* Tschitschérine, 1894 has been recorded from northern Xizang, Qinghai and Gansu Provinces and may also occur in far northern Sichuan. Its members differ from those of *Amara shalulishanica* in being smaller (body length less than 6.0 mm) and having the pronotum with its base more markedly punctate and lateral margins sinuate near the posterior angles. *Amara micans* Tschitschérine, 1894 is widespread in China, especially in Sichuan, and its members differ from those of *Amara shalulishanica* in being larger (body length greater than 8.0 mm in most individuals) and having the pronotum with lateral margins sinuate near the posterior angles and the front angles more distinctly extended forward of the anterior margin. Although some brachypterous specimens have been recorded from the Himalayan region, all *Amara micans* specimens from Sichuan examined are macropterous and have long metepisterna. The only other *Bradytulus* species with brachypterous members recorded from Sichuan is *Amara platynota* Hieke, 1994 (known from Daxueshan). Its members are larger (body length more than 8.0 mm), have a relatively wider body, broader head and darker legs, and its males have an S-shaped (in dorsal view) median lobe of the aedeagus (Hieke 1994, figs 88–89) and therefore cannot be confused with the new species.

#### Description.

**Figure 1.**
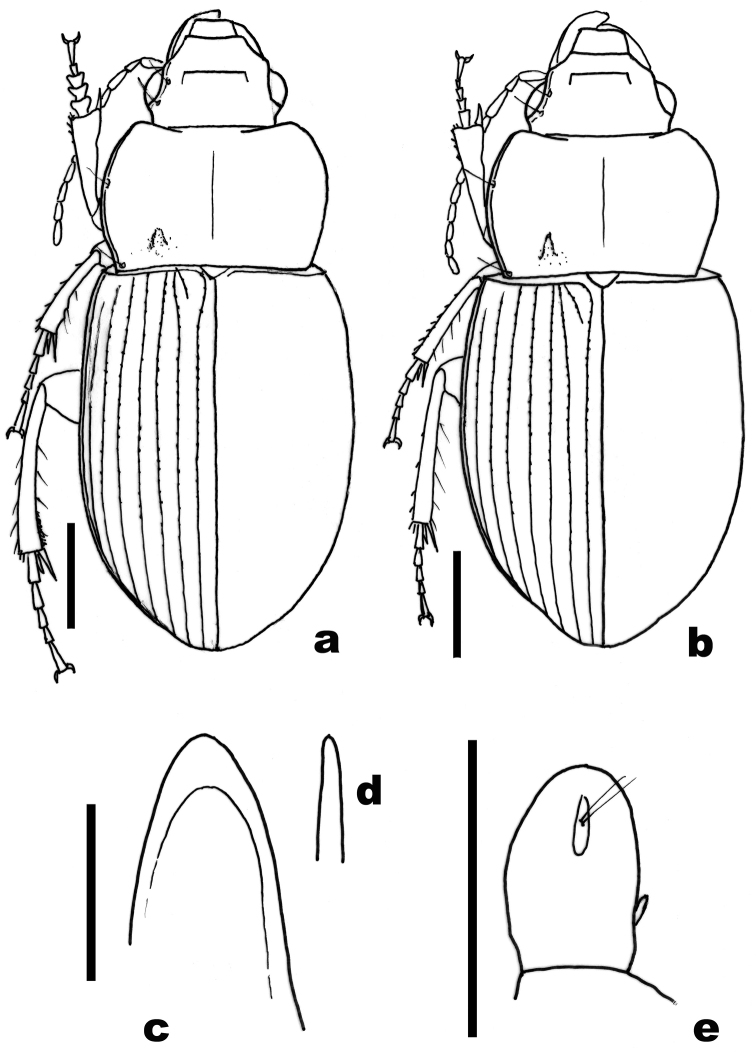
*Amara shalulishanica* sp. n. **a** dorsal habitus of holotype male **b** same of a paratype female **c** apex of the median lobe of aedeagus of holotype, dorsal view **d** same, left lateral view **e** gonostylus of female paratype, ventral view. Scale lines for **a** and **b** = 1.0 mm, for **c–e** = 0.5 mm.

Dorsal habitus as in Fig. 1a-b.Body length male 6.8–7.0 mm, female 6.6–7.0 mm. Color of body dark brown, antennae, palpi and legs reddish brown. Dorsal microscuplture comprised of isodiametric or nearly isodiametric sculpticells throughout, very faintly impressed on head in both sexes, more shallowly impressed on pronotum and elytra in males than in females; males with shinier dorsal luster than females.

Head smooth, broad, with distinct, hemispheric eyes.

Pronotum slightly transverse, with the greatest width slightly anterior to middle and posterior margin narrower than the base of elytra; lateral margins more rounded on anterior half, less arcuate or nearly straight in basal half; posterior margin slightly concave in middle; posterior angles distinct, slightly obtuse, narrowly rounded apically; anterior angles rounded, only slightly extended (about the diameter of the second antennomere) anteriorly beyond the front margin; inner basal foveae formed as short, deeply impressed longitudinal grooves; outer basal foveae absent; basal region with scattered, very fine punctures in and around inner basal foveae. Prosternum of male without a punctate fovea at middle; prosternal intercoxal process smoothly rounded apically, unmargined, asetose apically.

Pterothorax with metepisterna short, not longer than width across anterior margin.

Elytra with slightly curved sides and finely punctate striae; parascutellar striae short, located between striae 1 and 2 and extended from basal margin near base of stria 2 apicomedially toward stria 1; basal borders nearly straight, very slightly arched forward laterally; humeral teeth small but distinct and sharp; umbilicate setal series sparsely and unevenly spaced in the middle region; stria 7 without subapical setiferous pore punctures.

Hind wings short, reduced to a minute scale, hence adults flightless.

Legs with all femora bisetose; mesotibiae of males with a well-developed subapical medial tooth; metatibiae of males with a brush-like patch of setae ventrally in apical one-fourth.

Abdomen with venter only punctate laterally on the sternites 2 and 3. Male with one pair and female with two pairs of anal setiferous pore punctures at the apical margin of the last visible sternite.

Male genitalia with median lobe of aedeagus relatively broad, with apex rounded, apical lamella wider than long in dorsal view (Fig. 1c); right (longer) paramere without apical hook.

Female genitalia with gonostyli broadly oval (Fig. 1d), each with a short basolateral ensiform seta and an subapicoventral nematiform seta.

#### Etymology.

The species epithet, *shalulishanica*, is a Neolatin feminine adjective derived from the mountain range in which the type specimens were collected.

#### Geographical distribution.

Known only from the type locality, where adults were found in a high alpine meadow at 4500 m elevation; probably endemic to the central Shalulishan SSW of Litang.

#### Habitat distribution.

**Figure 2.**
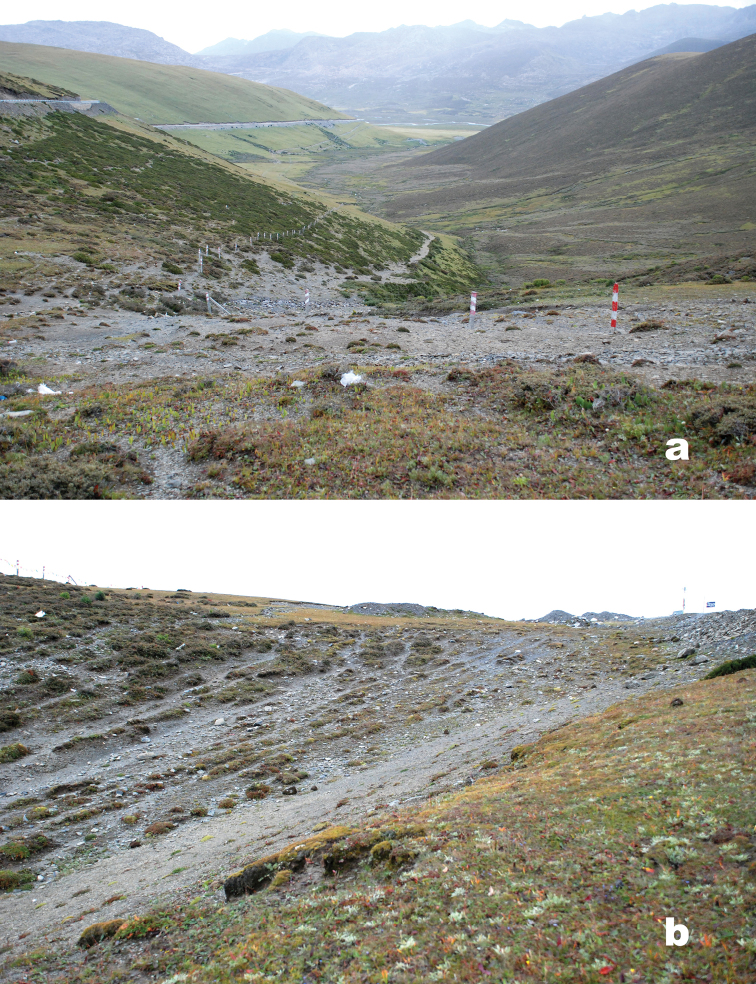
Photographs of type locality for *Amara shalulishanica* sp. n.at Haizishan Yakou [29.47366°N, 100.21921°E], Shalulishan, Zhuosang Township, Litang County, Sichuan Province, China, at an elevation of 4623 m. **a** view looking northeast from the Yakou (Pass) **b** view of area where the type series was collected.

All five specimens of the type series were collected within the area shown in Fig. 2b, under stones on barren substrate interspersed with areas of sparse, low, dry tundra vegetation. Stones under which beetles were found, even in more barren areas, often had accumulations of fine-scale vegetative debris, probably deposited there by wind. Specimens of the new species were collected along with those of two other *Amara* species, *Amara micans* Tschitschérine and *Amara litangensis* Hieke, 1994, in the same habitat.

#### Remarks.

All but one specimen (the smallest female) of the type series are teneral. Consequently, the color characteristics provided in the description are based solely on that one fully pigmented female specimen. However, because that specimen represents the far end (6.6 mm) of the size range of the type series (all others range between 6.8 and 7.0 mm in length), the holotype male was selected from among those other specimens despite its teneral condition. One consequence of this selection is that detailed structure of the internal sac of the male aedeagus, which typically includes more darkly pigmented or more heavily sclerotized features, cannot be distinguished in the holotype.

## Locality records for other *Amara* species

### Amara (Amara) kingdoni

Baliani, 1934

http://species-id.net/wiki/Amara_kingdoni

Amara (Amara) kingdoni Baliani, 1934b:243. Redescribed by Hieke 2002:647. Type material: Holotype male in BMNH. Type locality: Xizang (Tibet), SE Nagong, Shiuden Gompa, 13000 - 14000 ft.
Amara (Amara) sinica Hieke, 1997:247. Type material: Holotype male in ZMHB and 21 paratypes from various localities in Shaanxi, Sichuan and Qinghai Provinces in CFAC and ZIN. Type locality: China, Sichuan, Wa Shan (“Wanschan”). Synonymized by Hieke 2002:647.

#### Specimens examined.

Total of 14 specimens from the following localities: “CHINA, Yunnan, Shangrila County, Jiangtang Township, Shikashan, Napahai Houshan Yakou, 3590 m, 27.93404°N, 099.61287°E,”/“17 September 2007, Stop# 2007-042, D.H. Kavanaugh & H.B. Liang collectors”, 11 males and 1 female (CAS, IZCAS, ZMHB); “CHINA, Yunnan, Shangrila County, Xiaozhongdian Township, Tianbaoshan, 27.58517°N, 099.87586°E,”/ “3530 m, 18 September 2007, Stop# 2007-043, D.H. Kavanaugh & H.B. Liang collectors”, 2 males (CAS, IZCAS).

#### Geographical distribution.

Known only from China (Gansu, Qinghai, Sichuan, Xizang and Yunnan Provinces).

### Amara (Pseudoamara) birmana

Baliani, 1934

http://species-id.net/wiki/Amara_birmana

Amara (Amara) birmana Baliani, 1934a:189. Type material: Holotype female in BMNH, 1 paratype female in CBAL. Type locality: Burma [without specific locality, but probably from the mountains of northern Myanmar near the border with Yunnan Province, China]. Transferred to subgenus *Pseudoamara* Baliani by Hieke 2002:624.
Amara (Celia) yunnana Baliani, 1934a:193. Type material: Holotype male and 6 paratypes in BMNH. Type locality: China, Yunnan, Yunnan-fou. Synonymized by Hieke 1975:333.
Amara (Pseudoamara) beesoni Baliani, 1934a:190. Type material: Holotype male and allotype in BMNH, 4 paratypes in BMNH and MCSNG. Type locality: India, Assam, Shillong. Synonymized by Hieke 1975:285.

#### Specimens examined.

One male specimen (CAS) from the following locality: “CHINA, Yunnan, Lijiang County, Jiuhe Township, Laojunshan, 3510 m, 26.65079°N, 099.77449°E,”/ “21 September 2007, Stop# 2007-048, D.H. Kavanaugh & H.B. Liang collectors”.

#### Geographical distribution.

Known from China (Yunnan Province), India (Assam and Sikkim) and Myanmar.

### Amara (Celia) rupicola

C. Zimmermann, 1832

http://species-id.net/wiki/Amara_rupicola

Amara (Celia) rupicola C. Zimmermann, 1832:26. Type material: Number of syntypes not specified, whereabouts of type series unknown, probably lost; neotype not yet designated. Type locality: Russia, mountains of southern Siberia.
Amara (Celia) marginicollis A. Morawitz, 1863:259. Type material: Number of syntypes not specified, possibly several (4 localities given in original description), probably in ZIN; lectotype not yet designated. Type locality: Russia, Siberia, northern Baikal (locality listed in description). Synonymized by Tschitschérine 1899:164.
Amara ambigena Bates, 1878:716. Type material: Number of syntypes not specified, but probably only a holotype in BMNH (Andrewes 1930:24: “…Type seen…”). Type locality: India, Kashmir, Pangong Valley “Between Tanktze and Chagra” (Andrewes 1930:24). Synonymized by Andrewes 1930:24.
Amara (Celia) tiruka Andrewes, 1924:97. Type material: Lectotype male (designated by Hieke 1983:362) and 2 paralectotypes in BMNH. Type locality: India, Kashmir, Sonemarg and Ladakh, Leh. Synonymy suggested by Hieke 1981:203, formally synonymized by Hieke 1983:362.
Amara (Celia) faucium Andrewes, 1930:24. Type material: 7 syntypes in BMNH; the specimen cited as “Type” should be considered the holotype, specimens cited as “Cotype” should be considered as paratypes. Type locality: China, Xizang (southern Tibet), Jelep La, Phari, Tuna and Kampa Dzong. Synonymized by Hieke 1975:300.
Amara (Celia) rubroangulata Baliani, 1938:186. Type material: Holotype male and allotype in CBAL, 3 female paratypes in BMNH. Type locality: China: Sichuan and Xizang (eastern Tibet): “Lhodzong Poshö”, 12600 ft. Synonymized by Hieke 1981:202.
Amara (Amara) prochazkorum Jedlička, 1967:108. Type material: Holotype male in NMPC. Type locality: Mongolia, Karakorum village. Synonymized by Hieke 1999a:181.

#### Specimens examined.

Total of 13 specimens from the following localities: “CHINA, Sichuan, Kangding County, Xinduqiao Township, Liqi He, 3450 m, 30.02448°N, 101.52490°E,”/ “14 September 2007, Stop# 2007-035, D.H. Kavanaugh & H.B. Liang collectors”, 6 males and 4 females (CAS, IZCAS, ZMHB); “CHINA, Sichuan, Litang County, Gaocheng Township, Daxushan, Highway 318 at Km 3089, 4100 m, 30.03129°N, 100.33336°E,”/ “16 September 2007, Stop# 2007-040, D.H. Kavanaugh & H.B. Liang collectors”, 1 male and 2 females (CAS, IZCAS).

#### Geographical distribution.

Known from China (Gansu, Qinghai, Shaanxi, Sichuan, Xinjiang and Xizang Provinces), India (Jammu and Kashmir), Kazakhstan, Russia (from the Altai Mountains to Jakutia) and Turkmenistan(?).

### Amara (Xenocelia) mandarina

Baliani, 1932

http://species-id.net/wiki/Amara_mandarina

Amara (Bradytus) mandarina Baliani, 1932:15. Type material: Holotype male and 3 paratypes in MCSNG, 21 paratypes in BMNH, DEI, NMPC, RMNH, SNF and ZMHB. Type locality: China, Sichuan, Kangding (“Tatsienlu-Chiulung”). Erroneously synonymized with *Amara singularis* Tschitschérine by Hieke 1975:317; returned to species status and transferred to subgenus *Xenocelia* by Hieke 2001:104.

#### Specimens examined.

One male specimen (IZCAS) from the following locality: “CHINA, Sichuan, Kangding County, Xinduqiao Township, Gaoersishan, 1 km W of Yakou at Highway 318, 4245 m, 30.04988°N, 101.37485°E,”/ “15 September 2007, Stop# 2007-036, D.H. Kavanaugh & H.B. Liang collectors”.

#### Geographical distribution.

Known only from China (Sichuan and Yunnan Provinces).

### Amara (Pseudocelia) collivaga

Hieke, 1997

http://species-id.net/wiki/Amara_collivaga

Amara (Bradytus) collivaga Hieke, 1997:200. Type material: Holotype male in ZMHB, 1 paratype female in CHEI. Type locality: China, W. Sichuan, 30 km W of Kangding, 5000 m. Transferred to subgenus *Pseudocelia* by Hieke 2005:155.

#### Specimens examined.

Total of 6 specimens from the following locality: “CHINA, Sichuan, Kangding County, Lucheng Township, Zheduoshan at yakou on Highway 318, 4316 m, 30.07419°N, 101.80413°E,”/ “14 September 2007, Stop# 2007-033, D.H. Kavanaugh & H.B. Liang collectors”, 2 males and 4 females (CAS, IZCAS, ZMHB).

#### Geographical distribution.

Known only from China (Sichuan Province).

### Amara (Bradytus) chalciope

(Bates), 1891

http://species-id.net/wiki/Amara_chalciope

Leiocnemis chalciope Bates, 1891:71. Type material: Holotype male in BMNH. Type locality: China, Sichuan, Schneeberge bei Kangding (“Snowy Range, near Tatsienlu”), 13000 ft.
Amara (Niphobles) szetschuana Jedlička, 1934a:17. Type material: Holotype female in CJED. Type locality: China, Sichuan, Kangding (“Tatsienlu”). Transferred to subgenus *Bradytus* by Baliani 1937:176. Synonymized by Hieke 1983:361.

#### Specimens examined.

Total of 33 specimens from the following localities: “CHINA, Sichuan, Kangding County, Xinduqiao Township, Gaoersishan, 1 km W of yakou at Highway 318, 4245 m, 30.04988°N, 101.37485°E,”/ “15 September 2007, Stop# 2007-036, D.H. Kavanaugh & H.B. Liang collectors”, 3 males and 4 females; “CHINA, Sichuan, Luding County, Moxi Township, Hailuogou National Park, NE slope of Gongga Shan, 3035-3220 m, 29.57393°N, 101.99204°E to”/ “29.56721°N, 101.97979°E, 12 September 2007, Stop# 2007-031, D.H. Kavanaugh & H.B. Liang collectors”, 5 males and 2 females (CAS, IZCAS); “CHINA, Sichuan, Yajiang County, Hekou Township, Daxushan, Jianziwan Yakou at Highway 318, 4400-4450 m, 30.01203°N, 100.85983°E, ”/ “15 September 2007, Stop# 2007-037, D.H. Kavanaugh & H.B. Liang collectors”, 4 males and 5 females (CAS, IZCAS, ZMHB); “CHINA, Sichuan, Yajiang County, vicinity of Daoban work station 135 on road from Litang to Yajiang, 30.1399°N, 100.7068°E,”/ “4130 m, under stones in ditch along road adjacent to *Kobresia* meadow, 3 August 2006 B. Bartholomew collector”, 2 males and 8 females (CAS, IZCAS, ZMHB).

#### Geographical distribution.

Known from Bhutan and China (Fujian, Sichuan, Xizang and Yunnan Provinces).

### Amara (Bradytus) dissimilis

Tschitschérine, 1894

http://species-id.net/wiki/Amara_dissimilis

Amara (Bradytus) dissimilis Tschitschérine, 1894:404. Type material: Holotype male in ZIN. Type locality: China, Gansu, Ponggartang (“Thibet sept., Amdo, village Ndàmi”). [Note: The holotype was erroneously labeled “*Brad. dissors* Tschit. 1894 typ!” by Hieke (1999a:165)].
Amara (Bradytus) emmerichi Baliani, 1932:14. Type material: Holotype male (“type”) and one paratype in CBAL, additional paratypes in DEI (Döbler 1975:112), NMPC and ZMHB. Type locality: China, Sichuan, Kangding (“Tatsienlu-Chiulung”). Synonymized by Hieke 1999a:165.
Amara (Bradytus) lama Baliani, 1934c:110. Type material: Holotype female and 1 paratype in BMNH, 2 paratypes in CBAL. Type locality: SO.Tibet: Rong Tö Valley, 4000 - 7000 ft. Synonymized by Hieke 1997:225.
Amara (Bradytus) komala Jedlička, 1934b:116. Type material: Holotype female in CJED, 1 paratype female in CMEY. Type locality: China, Yunnan, Longchuan Jiang (“Soling-ho” Valley). Synonymized by Hieke 1995:297.
Amara (Bradytus) mera Jedlička, 1934b:116. Type material: Holotype female and 1 paratype female in CJED. Type locality: China, Yunnan, “Yunnan-fou”. Synonymized by Hieke 1995:297.

#### Specimens examined.

Total of 54 specimens from the following localitities: “CHINA, Yunnan, Lijiang County, Jiuhe Township, Laojunshan, 3500 m, 26.64210°N, 099.76745°E,”/ “20 September 2007, Stop# 2007-046A , D.H. Kavanaugh & H.B. Liang collectors”, 3 males and 3 females, (CAS, IZCAS); “CHINA, Yunnan, Shangrila County, Jiangtang Township, Shikashan, Napahai Houshan Yakou, 3590 m, 27.93404°N, 099.61287°E,”/“17 September 2007, Stop# 2007-042, D.H. Kavanaugh & H.B. Liang collectors”, 27 males and 16 females (CAS, IZCAS, ZMHB); “CHINA, Yunnan, Shangrila County, Xiaozhongdian Township, Tianbaoshan, 27.58517°N, 099.87586°E,”/ “ 3530 m, 18 September 2007, Stop# 2007-043, D.H. Kavanaugh & H.B. Liang collectors”, 1 male and 4 females (CAS, IZCAS).

#### Geographical distribution.

Known only from China (Gansu, Qinghai, Shaanxi, Sichuan, Xizang and Yunnan Provinces).

### Amara (Bradytus) sinuaticollis

A. Morawitz, 1863

http://species-id.net/wiki/Amara_sinuaticollis

Amara (Bradytus) sinuaticollis A. Morawitz, 1863:257. Type material: Number of syntypes not specified, at least two (male and female mentioned); 1 male and 1 female syntypes in ZIN. Type locality: Russia, “Ussuri”.

#### Specimens examined.

Total of 4 specimens from the following localities: “CHINA, Beijing, Huairou County, Shayu Township, Yugou Village, Xiangshui He, 218 m, 40.41614°N, 116.44386°E,”/ “26 October 2002, Stop # DHK-2002-052, D.H. Kavanaugh, P. E. Marek, & H.-B. Liang collectors”, 1 male and 1 female (CAS, IZCAS); “CHINA, Yunnan Province, Dali Prefecture, Yongping County, Qutong Township, 1600m, 25.42665°N, 99.52924°E, 25 June 2000, Stop #2000-005, D. H. Kavanaugh & Liang H.-B. collectors”, 1 male and 1 female (CAS, IZCAS).

#### Geographical distribution.

Known from China (Fujian, Gansu, Hebei, Shaanxi, Sichuan and Yunnan Provinces), Japan, Korea and Russia (Khabarovsky Krai and Primorsky Krai).

### Amara (Bradytus) pingshiangi

Jedlička, 1957

http://species-id.net/wiki/Amara_pingshiangi

Amara (Curtonotus) pingshiangi Jedlička, 1957:24. Type material: Lectotype female in CJED (Hieke 1990:238). Type locality: China: “Süd China: Pingshiang”, probably Jiangsu Province. Transferred to subgenus *Bradytus* by Hieke 1990:238.

#### Specimens examined.

One male specimen (CAS) from the following locality: “CHINA, Yunnan, Kunming City, Guandu District, Heilongtan, Kunming Institute of Botany Botanical Garden, 1945 m”/ 25.14035°N, 102.74107°E, 16-17 September 2002, Stop # DHK-2002-019, D.H. Kavanaugh & P.E. Marek collectors” [first record for Yunnan Province].

#### Geographical distribution.

Known only from China (Zhejiang, Fujian, Jiangsu and Sichuan Provinces).

### Amara (Bradytus) micans

Tschitschérine, 1894

http://species-id.net/wiki/Amara_micans

Amara (Bradytus) micans Tschitschérine, 1894:402. Type material: Lectotype (designated by Hieke 1973:111) and several paralectotypes in ZIN. Type locality: China, northern Qinghai Province, “Amdo-Plateau”, probably in the area around the confluence of the Blue and Yellow Rivers (west of Bayanharshan). Transferred to subgenus *Niphobles* by Hieke 1975:308 and to subgenus *Bradytulus* by Hieke 2003b:157.
Amara (Niphobles) splendens Andrewes, 1926:75. Type material: Holotype (“Type”) and paratype (“Cotype”) in BMNH, another paratype in FRSDD. Type locality: northern India, Himachal Pradesh (Kumaon District), Milam and Burphu in Gori Valley, 11500 ft. Synonymized by Hieke 1981:225.
Amara (Bradytus) pernix Csiki, 1929:457, replacement name for *Amara micans* Tschitschérine, 1894 (*nec*Letzner, 1852) [unnecessary change]. Synonymized by Hieke 1981:225.
Amara (Niphobles) jedlickai Baliani, 1932:158. Type material: 5 syntypes in MCSNG and MGFT (lectotype not yet designated). Type locality: China, Sichuan, Kangding (“Tatsienlu-Chiulung”). Synonymized by Hieke 1975:308.
Amara (Balianiia) vafra Lutshnik, 1935:267. Type material: Holotype male (“Monotypus”) in ZIN. Type locality: southern Mongolia, Gobi-Altai (“N.-Seite des Chines. Altai”). Synonymized by Hieke 1973:111.
Amara (Bradytus) eberti Jedlička, 1965:101. Type material: Holotype (“Type”) and 4 paratypes (“Cotype”) in ZSM, 2 additional paratypes in BMNH and 1 in ZMHB. Type locality: Nepal, Khumbu, Khumdzung. Synonymized by Hieke 1975: 298.
Amara (Bradytus) hellmichi Jedlička, 1965:102. Type material: 2 syntypes in ZSM and 1 in NMPC (lectotype not yet designated). Type locality: Nepal, Khumbu, Khumdzung. Synonymized by Hieke 1975:305.

#### Specimens examined.

Total of 11 specimens from the following localities: “CHINA, Sichuan, Litang County, Disan Township, Shalulishan, Haizishan Yakou, 29.47366°N, 100.21921°E,”/ “4623 m, 16 September 2007, Stop# 2007-041, D.H. Kavanaugh & H.B. Liang collectors”, 7 males and 3 females (CAS, IZCAS, ZMHB); “CHINA, Sichuan, Yajiang County, Hekou Township, Daxushan, Jianziwan Yakou at Highway 318, 4400-4450 m, 30.01203°N, 100.85983°E,”/ “15 September 2007, Stop# 2007-037, D.H. Kavanaugh & H.B. Liang collectors”, 1 female (CAS).

#### Geographical distribution.

Known from China (Gansu, Qinghai, Sichuan, Xizang and Yunnan Provinces), India (Jammu and Kashmir, Uttar Pradesh), Nepal and Pakistan.

### Amara (Reductocelia) daxueshanensis

Hieke, 2000

http://species-id.net/wiki/Amara_daxueshanensis

Amara (Reductocelia) daxueshanensis Hieke, 2000:118. Type material: Holotype male and 13 paratypes in ZMHB. Type locality: China, northern Yunnan, Zhongdian area, 3200–3300 m.

#### Specimens examined.

Total of 47 specimens from the following localities: “CHINA, Yunnan, Lijiang County, Jiuhe Township, Laojunshan, 3500 m, 26.64210°N, 099.76745°E,”/ “20 September 2007, Stop# 2007-046A , D.H. Kavanaugh & H.B. Liang collectors”, 1 female (CAS); “CHINA, Yunnan, Shangrila County, Jiangtang Township, Shikashan, Napahai Houshan Yakou, 3590 m, 27.93404°N, 099.61287°E,”/“17 September 2007, Stop# 2007-042, D.H. Kavanaugh & H.B. Liang collectors”, 3 males and 2 females (CAS, IZCAS); “CHINA, Yunnan, Shangrila County, Xiaozhongdian Township, Tianbaoshan, 27.58517°N, 099.87586°E,”/ “3530 m, 18 September 2007, Stop# 2007-043, D.H. Kavanaugh & H.B. Liang collectors”, 19 males and 22 females (CAS, IZCAS, ZMHB).

#### Geographical distribution.

Known only from China (Yunnan Province).

### Amara (Reductocelia) dequensis

Hieke, 1999

http://species-id.net/wiki/Amara_dequensis

Amara (Reductocelia) dequensis Hieke, 1999b:347. Type material: Holotype male and 13 paratypes in ZMHB. Type locality: China, northern Yunnan, Zhongdian area, 3200–3300 m.

#### Specimens examined.

One male specimen (IZCAS) from the following locality: “CHINA, Sichuan, Batang County, pass between Ciwu and Zhong Xinrong on road from Derong to Batang, 30.1399°N, 100.7068°E,”/ “4130 m, under stones in oak scrub slope adjacent to *Kobresia* meadow, 29 July 2006 B. Bartholomew collector”.

#### Geographical distribution.

Known only from China (Sichuan and Yunnan Provinces).

### Amara (Reductocelia) litangensis

Hieke, 1994

http://species-id.net/wiki/Amara_litangensis

Amara (Bradytulus) litangensis Hieke, 1994:315. Type material: Holotype male and 8 paratypes in CSCI, 5 paratypes in ZMHB and 2 paratypes in CWRA. Type locality: China, Sichuan, 10 km S of Litang, 4000 m. Transferred to subgenus *Reductocelia* by Hieke 1999b:348.

#### Specimens examined.

Total of 18 specimens from the following locality: “CHINA, Sichuan, Litang County, Disan Township, Shalulishan, Haizishan Yakou, 29.47366°N, 100.21921°E,”/ “4623 m, 16 September 2007, Stop# 2007-041, D.H. Kavanaugh & H.B. Liang collectors”, 5 males and 13 females (CAS, IZCAS, ZMHB).

#### Geographical distribution.

Known only from China (Sichuan Province).

### Amara (Reductocelia) stricticeps

Baliani, 1932

http://species-id.net/wiki/Amara_stricticeps

Amara (Celia) stricticeps Baliani, 1932:7. Type material: Holotype male and allotype in CBAL, 1 paratype in DEI (Döbler 1975:142) and also in SNF. Type locality: China, Sichuan, Kangding (“Tatsienlu-Chiulung”). Transferred to subgenus *Leiromorpha* by Baliani 1937:176 and to subgenus *Reductocelia* by Hieke 1999b:356.

#### Specimens examined.

Total of 58 specimens from the following localities: “CHINA, Sichuan, Kangding County, Lucheng Township, Zheduoshan at yakou on Highway 318, 4316 m, 30.07419°N, 101.80413°E,”/ “14 September 2007, Stop# 2007-033, D.H. Kavanaugh & H.B. Liang collectors”, 1 male (CAS); “CHINA, Sichuan, Kangding County, Xinduqiao Township, Gaoersishan, 1 km W of yakou at Highway 318, 4245 m, 30.04988°N, 101.37485°E,”/ “15 September 2007, Stop# 2007-036, D.H. Kavanaugh & H.B. Liang collectors”, 23 males and 16 females (CAS, IZCAS, ZMHB); “CHINA, Sichuan, Litang County, Gaocheng Township, Daxushan, Highway 318 at Km 3089, 4100 m, 30.03129°N, 100.33336°E,”/ “16 September 2007, Stop# 2007-040, D.H. Kavanaugh & H.B. Liang collectors”, 3 males and 1 female (CAS, IZCAS); “CHINA, Sichuan, Yajiang County, Hekou Township, Daxushan, Jianziwan Yakou at Highway 318, 4400-4450 m, 30.01203°N, 100.85983°E,”/ “15 September 2007, Stop# 2007-037, D.H. Kavanaugh & H.B. Liang collectors”, 10 males and 3 females (CAS, IZCAS, ZMHB).

#### Geographical distribution.

Known only from China (Sichuan Province).

### Amara (Curtonotus) kangdingensis

Hieke, 1997

http://species-id.net/wiki/Amara_kangdingensis

Amara (Curtonotus) kangdingensis Hieke, 1997:222. Type material: Holotype male and 1 paratype male in CHEI**,** 1 paratype male in ZMHB. Type locality: China, Sichuan, pass 30 km W of Kangding, 4000–4200 m.

#### Specimens examined.

Total of 13 specimens from the following localities: “CHINA, Sichuan, Kangding County, Lucheng Township, Zheduoshan at yakou on Highway 318, 4316 m, 30.07419°N, 101.80413°E,”/ “14 September 2007, Stop# 2007-033, D.H. Kavanaugh & H.B. Liang collectors”, 2 males and 3 females (CAS, IZCAS, ZMHB).

#### Geographical distribution.

Known only from China (Sichuan Province).

### Amara (Curtonotus) macronota

Solsky, 1875

http://species-id.net/wiki/Amara_macronota

Curtonotus nitens Putzeys, 1866:234 (*nec*Sturm, 1825). Type material: Holotype, female, in CCHA. Type locality: northern China [without specific locality (“Chine boréale”)].
Curtonotus macronotus Solsky, 1875:265. Type material: Holotype, in ZIN (Tschitschérine 1894:386). Type locality: Russia, Primorsky Krai, Suyfun River at «Nikolskoje». Synonymized by Tschitschérine 1894:385.
Amara (Curtonotus) jureceki Jedlička, 1957:29. Type material: Number of syntypes not specified, but probably only the holotype (based on text of description), in CJED.- Type locality: Russia, Primorsky Krai, Vladivostok. Synonymized by Lafer 1989:180.
Amara (Curtonotus) ovalipennis Jedlička, 1957:30. Type material: Holotype, male, in CJED. Type locality: Japan, Kyoto. Regarded as a subspecies, *Amara macronota ovalipennis* Jedlička, by Morita 1987:70. Synonymized by Hieke 1995:322.

#### Specimens examined.

Two female specimens (CAS, IZCAS) from the following locality: “CHINA, Beijing, Wuling Mountains, Miyun County, Xinchangzi Township, Xiakou Village, small branch of Andamu He, 415 m,”/ “40.65278°N, 117.34069°E, 27 October 2002 Stop # DHK-2002-056, D.H. Kavanaugh, P. E. Marek, & H.-B. Liang collectors”.

#### Geographical distribution.

Known from China (Gansu, Shaanxi, Sichuan and Yunnan Provinces), Japan, Korea and Russia (Primorsky Krai and Khabarovsky Krai).

## Discussion

The diverse carabid beetle fauna of China and adjacent areas remains relatively poorly sampled and many new species are discovered in and described from the region each year. The geographical distributions of virtually all species in the fauna are still poorly known, and this is certainly true for the Chinese *Amara* species. Despite this incomplete knowledge of the fauna, however, the *Amara* species recorded from Yunnan and Sichuan Provinces can be grouped, at least tentatively, into five different distributional types. The species on which we have reported here are listed below according to their apparent distributional type.

1. *Widespread eastern and central Palaearctic species*. These include species occurring in Russia (from the Altai to the Amur region, from Yakutia to the Mongolian border), Mongolia, and northern China (rarely as far south as the Himalaya): *Amara rupicola*.

2. *Macropterous eastern Palaearctic species*. These include species occurring in central Japan, the Ussuri region of the Russian Far East, Korea, eastern Mongolia, and China (excluding the west but often including Taiwan): *Amara sinuaticollis* and *Amara macronota*.

3. *Macropterous species widespread in China*. These include species occurring in several Chinese provinces, and in many cases also in regions of the neighboring Himalayan countries of India, Nepal, Bhutan, and Myanmar: *Amara kingdoni*, *Amara mandarina*, *Amara chalciope*, *Amara dissimilis*, *Amara pingshiangi*,and *Amara micans*.

4. *Macropterous Himalayan species*. These include species occurring at least in the central and eastern parts of this region, and often also in Yunnan, less commonly also in southern Sichuan: *Amara birmana*.

5. *Apterous endemic Chinese species*. These include species occurring only in small areas, mainly in isolated mountain ranges that occupy only part of one province or border areas linking adjacent parts of two provinces: *Amara collivaga*, *Amara shalulishanica* sp. n., *Amara daxueshanensis*, *Amara dequensis*, *Amara litangensis*, *Amara stricticeps*, and *Amara kangdingensis*.

As additional sampling throughout the region continues, particularly in remote areas not yet explored, the ranges of known and additional, still undiscovered species will become better known. The appropriateness of recognizing these different distributional types for characterizing the geographical ranges of different *Amara* species, as well as other species of the regional fauna, will be tested by these future findings.

## Supplementary Material

XML Treatment for Amara (Bradytulus) shalulishanica

XML Treatment for Amara (Amara) kingdoni

XML Treatment for Amara (Pseudoamara) birmana

XML Treatment for Amara (Celia) rupicola

XML Treatment for Amara (Xenocelia) mandarina

XML Treatment for Amara (Pseudocelia) collivaga

XML Treatment for Amara (Bradytus) chalciope

XML Treatment for Amara (Bradytus) dissimilis

XML Treatment for Amara (Bradytus) sinuaticollis

XML Treatment for Amara (Bradytus) pingshiangi

XML Treatment for Amara (Bradytus) micans

XML Treatment for Amara (Reductocelia) daxueshanensis

XML Treatment for Amara (Reductocelia) dequensis

XML Treatment for Amara (Reductocelia) litangensis

XML Treatment for Amara (Reductocelia) stricticeps

XML Treatment for Amara (Curtonotus) kangdingensis

XML Treatment for Amara (Curtonotus) macronota
